# P43 Determining the antibiotic resistance phenotypes of *Klebsiella pneumoniae* infections in children aged from 0 to 59 months in the health district of Koutiala in Mali from August 2023 to March 2024

**DOI:** 10.1093/jacamr/dlaf118.050

**Published:** 2025-07-14

**Authors:** Diawara Moussa Karim, Traore Ibrahim, Koné Souleymane

**Affiliations:** Koutiala Paediatric Medico-Nutritional Project of Médecins Sans Frontières France, Mali; Koutiala Paediatric Medico-Nutritional Project of Médecins Sans Frontières France, Mali; Department of Study and Research in Faculty of Science and Technologie, Bamako, Mali

## Abstract

**Background:**

In the Koutiala region of Mali, preventing and effectively treating klebsiella pneumoniae infections children aged 0 to 59 months requires ongoing clinical research. *Klebsiella pneumoniae* is an enterobacteria responsible for many serious infections such as pneumoniae, especially nosocomial. Antibiotic resistance is a major problem and presents a challenge for the treatment of *K. pneumoniae* infections.

**Objectives:**

To study the antibiotic resistance phenotypes of *K. pneumoniae* 2024.

**Methods:**

Human blood and urine samples were received and grown in the laboratory to isolate strains and determine resistance phenotypes

**Results:**

On the 2771 samples treated during our study, there were 623 positive or 22%. The most frequently found germs in the blood and urine were *Escherichia coli*, *Salmonella* spp., *K. pneumoniae* and *Streptococcus pneumoniae*. The distribution of *K. pneumoniae* strains 4% (25 strains) for positive samples. That is 64% in the blood and 36% in the urine. The number of isolated strains of *K. pneumoniae* was in favour of the male sex with 56% and 44% for the female sex. The number of isolate strains of *K. pneumoniae* was 64% for infants, 32% for children, and 4% for newborns. The services that recorded the most strains were the nutrition services and hospital paediatrics. For the future of patients 12% died during our study. Regarding antibiotic resistance, there was ampicillin, the combination trimethoprim+ sulfamethoxazole, ceftriaxone, amoxicillin + clavulanic acid, cefoxitin; aztreonam and cefepime. The most sensitive antibiotics were amikacin, cefotaxime, ertapenem and meropenem.

**Conclusions:**

This study showed that 25 strains of *K. pneumoniae* were isolated, including 20 strains of BMR among which there were 15 strains of BLSE and 5 strains of suspected carbapenemases.

